# Chondroitin Sulfate-E Is a Negative Regulator of a Pro-Tumorigenic Wnt/Beta-Catenin-Collagen 1 Axis in Breast Cancer Cells

**DOI:** 10.1371/journal.pone.0103966

**Published:** 2014-08-04

**Authors:** Catherine M. Willis, Michael Klüppel

**Affiliations:** 1 Stanley Manne Children’s Research Institute, Chicago, Illinois, United States of America; 2 Department of Pediatrics, Robert H. Lurie Comprehensive Cancer Center, Feinberg School of Medicine, Northwestern University, Chicago, Illinois, United States of America; 3 Robert H. Lurie Comprehensive Cancer Center, Feinberg School of Medicine, Northwestern University, Chicago, Illinois, United States of America; Virginia Commonwealth University, United States of America

## Abstract

Expression of the glycosaminoglycan chondroitin sulfate-E (CS-E) is misregulated in many human cancers, including breast cancer. Cell-surface associated CS-E has been shown to have pro-tumorigenic functions, and pharmacological treatment with exogenous CS-E has been proposed to interfere with tumor progression mediated by endogenous CS-E. However, the effects of exogenous CS-E on breast cancer cell behavior, and the molecular mechanisms deployed by CS-E are not well understood. We show here that treatment with CS-E, but not other chondroitin forms, could interfere with the invasive protrusion formation and migration of breast cancer cells in three-dimensional organotypic cultures. Microarray analysis identified transcriptional programs controlled by CS-E in these cells. Importantly, negative regulation of the pro-metastatic extracellular matrix gene *Col1a1* was required for the anti-migratory effects of exogenous CS-E. Knock-down of *Col1a1* gene expression mimics the effects of CS-E treatment, while exposing cells to a preformed collagen I matrix interfered with the anti-migratory effects of CS-E. In addition, CS-E specifically interfered with Wnt/beta-catenin signaling, a known pro-tumorigenic pathway. Lastly, we demonstrate that *Col1a1* is a positively regulated target gene of the Wnt/beta-catenin pathway in breast cancer cells. Together, our data identify treatment with exogenous CS-E as negative regulatory mechanism of breast cancer cell motility through interference with a pro-tumorigenic Wnt/beta-catenin - Collagen I axis.

## Introduction

Breast cancer is one of the most commonly diagnosed and most invasive cancers in women, and it is the second leading cause of death in women in the U.S. [1]. Targeting molecules of the tumor microenvironment has become an active area of research for cancer treatment [2]–[4]. One component of the tumor microenvironment is the glycosaminoglycan chondroitin sulfate (CS). CS biosynthesis and sulfation balance is tightly controlled and of critical importance in development and disease [5]–[14]. Cell type-specific sulfation balance is influenced by growth factor signaling and in turn can control cellular signaling pathways [7]–[11], [13], [14]. The specific sulfation pattern of CS chains dictates its function and binding affinities [7], [9], [15].

Several studies have shown potential roles of CS and CS proteoglycans in tumor biology. A marked increase of CS and CS proteoglycans has been observed in many human solid tumors, including prostate cancer, ovarian adenocarcinomas, colon cancer, and breast cancer [16]–[21]. Recent work by our laboratory and others suggests that endogenous CS molecules have distinct temporal functions during breast cancer progression: an anti-metastatic function in primary tu­mor tissue [11], but a pro-metastatic role during the interaction of circulating cancer cells with endothelial cells (extravasation) [22]. Higher amounts of the double sulfated CS-E unit were found on a highly metastatic mouse osteosarcoma cell line, when compared to the non-metastatic parental tumor line [23]. Tissue colonization experiments demonstrated that preincubation of these metastatic tumor cells with an antibody against endogenous CS-E, or administration of exogenous CS-E together with tumor cells, could interfere with colonization of the liver [23]. Similar results were obtained with mouse lung carcinoma cells in a different study [24]. Breast cancer cell surface CS-E has been shown to bind P-selectin on endothelial cells *in vitro*, suggestive of a potential role of CS-E-proteoglycans in tumor cell adhesion to the endothelium during extravasation of the circulating tumor cells to target organs [20].

Despite these insights, the molecular mechanisms through which CS-E controls cancer cell behavior are not well understood. *In vitro* studies have identified cellular signaling pathways regulated by CS-E [25]. We and others have previously shown that exogenous CS-E can inhibit Wnt/beta-catenin signaling in fibroblasts, and can specify Wnt/beta-catenin signaling thresholds for distinct transcriptional and biological readouts [13]. The Wnt/beta-catenin pathway is of critical importance in many developmental processes [26]–[28], and also has known pro-tumorigenic and pro-metastatic functions in many human cancers [27], including breast cancer [29], [30].

Here, we set out to investigate the roles of CS-E in the behavior of two murine mammary carcinoma cell lines. We show that exogenous treatment with CS-E, but not other chondroitin sulfation forms, can drastically interfere with the invasive protrusion formation of breast cancer cells when grown in 3D Matrigel culture. This was in part to due to the ability of CS-E to negatively regulate cell migration. We further demonstrate by microarray analysis that CS-E differentially regulated the expression of several genes, including the pro-metastatic extracellular matrix genes *Col1a1* and *Col6a2* [28], [30]. Knock-down of *Col1a1* gene expression mimics the effects of CS-E treatment, while exposing cells to a preformed collagen I matrix interfered with the anti-migratory effects of CS-E. We go on to show that CS-E negatively regulates Wnt/beta-catenin signaling, a known pro-tumorigenic pathway, and that *Col1a1* is a positively regulated target gene of the Wnt/beta-catenin pathway in breast cancer cells. Together, our data demonstrate that CS-E could negatively regulate *Col1a1* gene expression through inhibition of Wnt/beta-catenin signaling, which in turn led to decreased breast cancer cell motility. These data identify a novel CS-based control mechanism for a Wnt/beta-catenin-collagen I pro-tumorigenic axis, and provide evidence for a potential therapeutic use of CS-E as an inhibitor of Wnt/beta-catenin signaling in breast cancer.

## Materials and Methods

### Cell lines, reagents, and treatments

Mouse breast cancer cell lines EMT6 and 4T1, as well as L-cells and L-Wnt3a-cells were obtained from ATCC, USA. All cell lines were maintained in Dulbecco’s Modified Eagle’s Medium (DMEM) (Invitrogen) containing 10% FBS (Hyclone). BD Growth Factor Reduced Matrigel, BD Matrigel Matrix Growth Factor Reduced, and BD BioCoat Control 8.0 micrometer PET Membrane 24-well Cell Culture Inserts were purchased from BD Biosciences, USA. ‘Super Special Grade’ C4S, C6S, CS-D, and CS-E, purified using Schiller’s column chromatographic method, were obtained from Seikagaku/The Associates of Cape Cod, USA. All CS preparations were received as lyophilized Na-salts, reconstituted in H_2_O, aliquoted, and stored at –20°C. Biological sources for the different CS types are as follows: C4S: sturgeon notochord; C6S: shark cartilage; CS-D: shark cartilage; CS-E: squid cartilage. Chondroitinase ABC (protease-free) was purchased from Seikagaku/The Associates of Cape Cod, USA. Bovine Collagen, Type I was purchased from BD Biosciences, USA. Wnt Antagonist I, IWR-1-*endo* was purchased from Calbiochem (EMD Millipore), San Diego, CA.

### 3D matrigel cell culture and immunofluorescence

Cells were plated on top of growth factor reduced Matrigel as previously described [31], in the presence or absence of C4S, C6S, CS-D, or CS-E for a total of six days with bi-daily changing of treatment, and were assessed by live phase contrast microscopy. Chondroitinase ABC (100 mU/ml f.c.) digestion of CS-E was performed for 2 hours at 37°C prior to addition to Matrigel cultures. Invasive protrusions were counted as a measure of invasive behavior on EMT6 3D structures in at least 10 fields per condition. Immunofluorescence was performed as previously described [31]. For EdU proliferation studies, the Click-iT EdU kit (Invitrogen, USA) was used according to the manufacturer’s instructions. EdU was added to 3D cultures on Day 6, one hour prior to fixation. For TUNEL labeling of cells undergoing apoptosis, an In Situ Cell Death Detection Kit, Fluorescein (Roche, USA) was used according to the manufacturer’s instructions. All 3D structure immunofluorescence images were obtained from the Zeiss LSM510 Meta using Zen 2009 software.

### Transwell migration and invasion assays

Assays were performed according to manufacture’s protocol: EMT6 or 4T1 cells (3×10^4^) were plated onto the 24-well transwell migration control inserts (BD Biosciences; 8 µm pore size) or BD BioCoat invasion chambers coated with growth factor reduced Matrigel (BD Biosciences). Cells were incubated in either serum-free DMEM control conditions or with 100 microgram/ml CS-E in the upper chamber, and DMEM/10%FBS in the lower chamber, and incubated for 24 hours. Cells that adhered to the bottom of insert were fixed using 4% PFA and stained with DAPI. All cells that migrated or invaded were quantified. Invasive cells are displayed as a measure of percent invasion, which is corrected for by the number of cells that migrated in the same condition in the control inserts. For transwell migration assays coated with Collagen I, EMT6 or 4T1 cells (2.5×10^5^) were plated on migration control inserts from BD Biosciences that were uncoated or coated with Bovine Collagen, Type 1 (500 microgram/ml) and treated as described above. Cells were incubated for 8 hours. The average number of cells per field in at least 8 fields per condition was calculated.

### Gene expression profiling by microarray

EMT6 cells grown in 3D Matrigel culture, as described above, were treated in control conditions or with CS-E at 100 microgram/ml, and harvested after 6 days for RNA. RNA was extracted using TRI Reagent (Fisher Scientific, USA) and the RNeasy Mini Kit (Qiagen, USA) according to the manufacturer’s instructions. Subsequently, gene expression profiling was performed at the Northwestern University Center for Genetic Medicine Genomics Core Facility, utilizing Illumina Mouse-8 Gene Expression Chips on an Illumina iScan platform. Genes that were up- or downregulated 1.8-fold or higher were assembled into the final list of target genes. Microarray datasets have been submitted to the ArrayExpress repository under accession number E-MTAB-2645.

### Quantitative real time PCR (qRT-PCR)

RNA was prepared using TRI Reagent according to the manufacturer’s protocol. Subsequently, 1.5 micrograms of RNA were reversed transcribed using Reverse Transcriptase (Promega, USA), followed by real time amplification using Power-SYBR Green PCR Master Mix (Applied Biosystems, USA) on an Applied Biosystems 7500 Real Time PCR platform in 15 microliter reactions using an annealing temperature of 60°C. The following primer pairs were used (5′ to 3′): *HPRT,* AAACAATGCAGACTTTGCTTTCCTTGG and GGTCCTTTTCACCAGCAAGCTTGCG; *Col6a2,* ATCGTGTGTCCAGAACTTCCC and AGCAGGAAGACAATGTCCACG; *Notum*, ACTGCGTGGTACACTCAAGG and GACGTCCGTCCAATAGCTCC; *Mmp13*, GCCATTACCAGTCTCCGAGG and CAGCATCCACATGGTTGGGA; *Saa3,* TGGGAGTTGACAGCCAAAGA and GCATCATAGTTCCCCCGAGC; *Calr,* GCATAGGCCTCATCATTGGT and AATACTCCCCCGATGCAAAT; *Col1a1,* CTCCTCTTAGGGGCCACTGCC and GGGTTTCCACGTCTCACCATTGG; *Ccl7*, TCCCTGGGAAGCTGTTATCTTCA and AGGCTTTGGAGTTGGGGTTT; *Klf6,* CGACATGGATGTGCTCCCAA and CAACTCCAGGCAGGTCTGTT; *Trps1,* CGAGACACTACAGGAGAGCAC and CCCTCTTCGCCATTAGCAGT.

### siRNA knockdown of Col1a1

The Col1a1 siRNA pool (siCol1a1) was purchased from Santa Cruz Biotechnology (sc-44044). The non-targeting control siRNA is targeting the luciferase gene (siCon), and was purchased from Thermo Fisher Scientific. EMT6 or 4T1 cells were seeded at a density of 2×10^5^ cells/well of a 12 well plate. After 24 hours the cells were transfected with 0.5 micrograms of siCol1a1 or siCon using Lipofectamine 2000 (Life Technologies) and analyzed after 24 hours.

### Immunoblotting

EMT6 or 4T1 cells were harvested for protein lysates for immunoblotting as described in [31]. List of antibodies: anti-collagen I (ab34710, abcam), anti-beta-catenin (sc-7963, Santa Cruz Biotechnology), anti-alpha-tubulin (sc-8935, Santa Cruz Biotechnology), anti-phospho-LRP6 (#2568, Cell Signaling Technology), and anti-LRP6 (#3395, Cell Signaling Technology). Densitometry was determined by the ImageJ program.

### Immunofluorescence

Non-confluent monolayers of EMT6 or 4T1 cells were incubated in L-CM or Wnt3a-CM, with or without 100 microgram/ml CS-E. Cells were fixed in 4% PFA, permeabilized using 0.2% TX-100, and immunofluorescence staining was performed according to standard methods. Primary antibody was anti-beta-catenin (sc-7963, Santa Cruz Biotechnology), followed by Alexa-488-conjugated secondary antibody (Invitrogen, USA), and DAPI as a nuclear stain.

### TOPFLASH reporter assay

EMT6 or 4T1 cells were plated at a density of 3×10^4^ cells per well of a 48 well plate. After 24 hours they were transfected with firefly TOPFLASH and Renilla luciferase transfection control reporter constructs, using linear PEI (MW: 25,000; Polysciences, USA; PEI/DNA ratio of 5∶1) as a transfection reagent. Three hours post-transfection the cells were stimulated with Wnt3a-CM for 24 hours. Dual luciferase assays were performed according to manufacturer’s instructions (Promega, USA; Biotium, USA).

### Statistical analysis

All experiments were repeated at least three times and p-values were obtained using an unpaired student’s t-test.

## Results

### CS-E inhibits invasive protrusion formation of EMT6 cells grown in 3D culture

**Figure 1:**
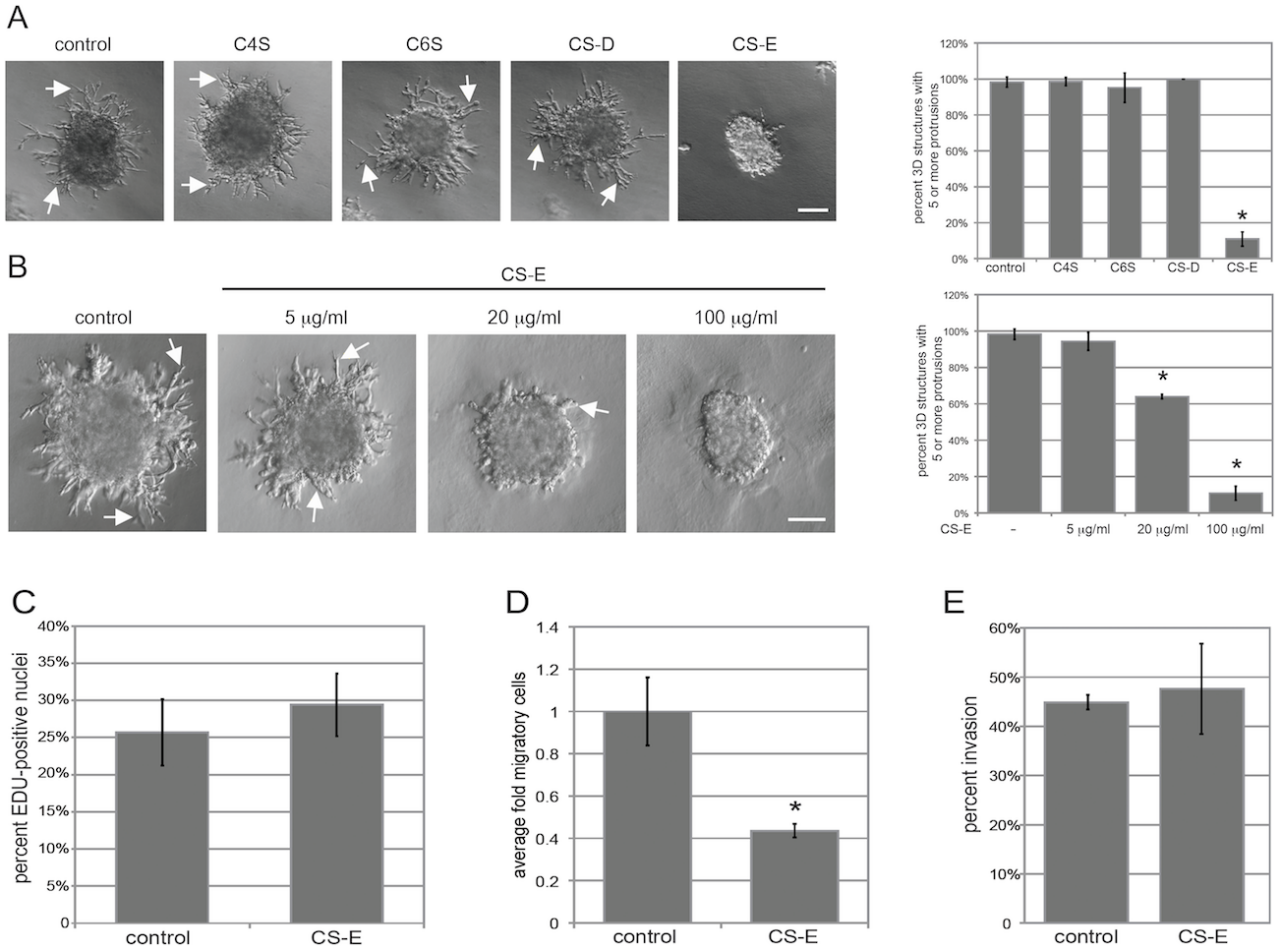
CS-E reduces 3D invasive protrusion formation and migratory behavior of EMT6 cells. (**A**) EMT6 cells grown in 3D on-top Matrigel assays were treated with 100 microgram/ml C4S, C6S, CS-D, CS-E, or no treatment control conditions for 6 days, and assessed by live phase contrast microscopy (scale bar, 100 micrometer). At day 6, the percent of 3D structures containing 5 or more invasive protrusions were quantified for each condition. Arrows represent invasive protrusions on 3D structures. (**B**) Inhibition of invasive protrusion formation by CS-E treatment is concentration dependent. EMT6 cells were treated in control conditions or with increasing amounts of CS-E for 6 days in 3D on-top Matrigel conditions. (**C**) EMT6 cell 3D cultures were grown in control conditions or with 100 microgram/ml CS-E for 6 days with EdU addition 1 hour prior to fixation for detection of proliferating cells by confocal microscopy. (**D, E**) Invasion/migration transwell assays. EMT6 cells were plated on migration control inserts (**D**), or on BD BioCoat invasion chambers coated with growth factor reduced Matrigel (**E**) and treated with or without 100 microgram/ml of CS-E for 24 hours. Total cell counts show that CS-E treatment reduced EMT6 cell migration by 2.3 fold, but did not change cell invasion. (n = 6). (*p<0.01).

To get an initial understanding of the potential roles of the differentially sulfated CS molecules in breast cancer cells, we set out to characterize the effects of treatment with exogenous C4S, C6S, CS-D and CS-E on EMT6 cells grown in a well-established organotypic three dimensional (3D) on-top Matrigel assay (Figure 1) [31]. EMT6 cells are a highly metastatic cell line [32], and therefore display invasive activity when grown on growth factor reduced Matrigel in an on-top 3D assay, with many invasive protrusions emanating from the core of the 3D structures. Untreated control cultures showed multiple invasive protrusions at day 6 of culture (Figure 1A). Treatment with C4S, C6S, or CS-D at 100 microgram/ml did not change the invasive characteristics of EMT6 cells (Figure 1A). However, treatment with CS-E at 100 microgram/ml severely inhibited the invasive protrusion formation of EMT6 cells (Figure 1A). Quantification demonstrated that nearly 100% of EMT6 3D structures grown in control conditions (no treatment) had 5 or more invasive protrusions per structure. This was not altered by treatment with C4S, C6S, or CS-D. In contrast, only about 10% of EMT6 3D structures treated with CS-E had 5 or more invasive protrusions. This specific effect of CS-E was dose dependent (Figure 1B). Treatment with 5 microgram/ml CS-E resulted in 94% of EMT6 3D structures having 5 or more invasive protrusions, 20 microgram/ml CS-E resulted in 64% of structures having 5 or more invasive protrusions, and treatment with 100 microgram/ml CS-E led to 11% of EMT6 3D structures having 5 or more invasive protrusions. To confirm that this effect is not due to other factors present in our CS-E preparation, we performed enzymatic digestion of CS-E with Chondroitinase ABC for 2 hours prior to treatment of cultures. This digestion eliminated the inhibitory effect of CS-E on protrusion formation, demonstrating that inhibition of protrusion formation was dependent on intact CS-E (Figure S1). Increasing the concentration of C4S, C6S or CS-D five-fold to 500 microgram/ml resulted in only minor inhibitory effects on protrusion formation (Figure S1). These results show that CS-E can dramatically reduce invasiveness of breast cancer cells *in vitro*, while other CS forms only mildly affect protrusion formation at much higher concentrations.

### CS-E decreases EMT6 cell motility

**Figure 2:**
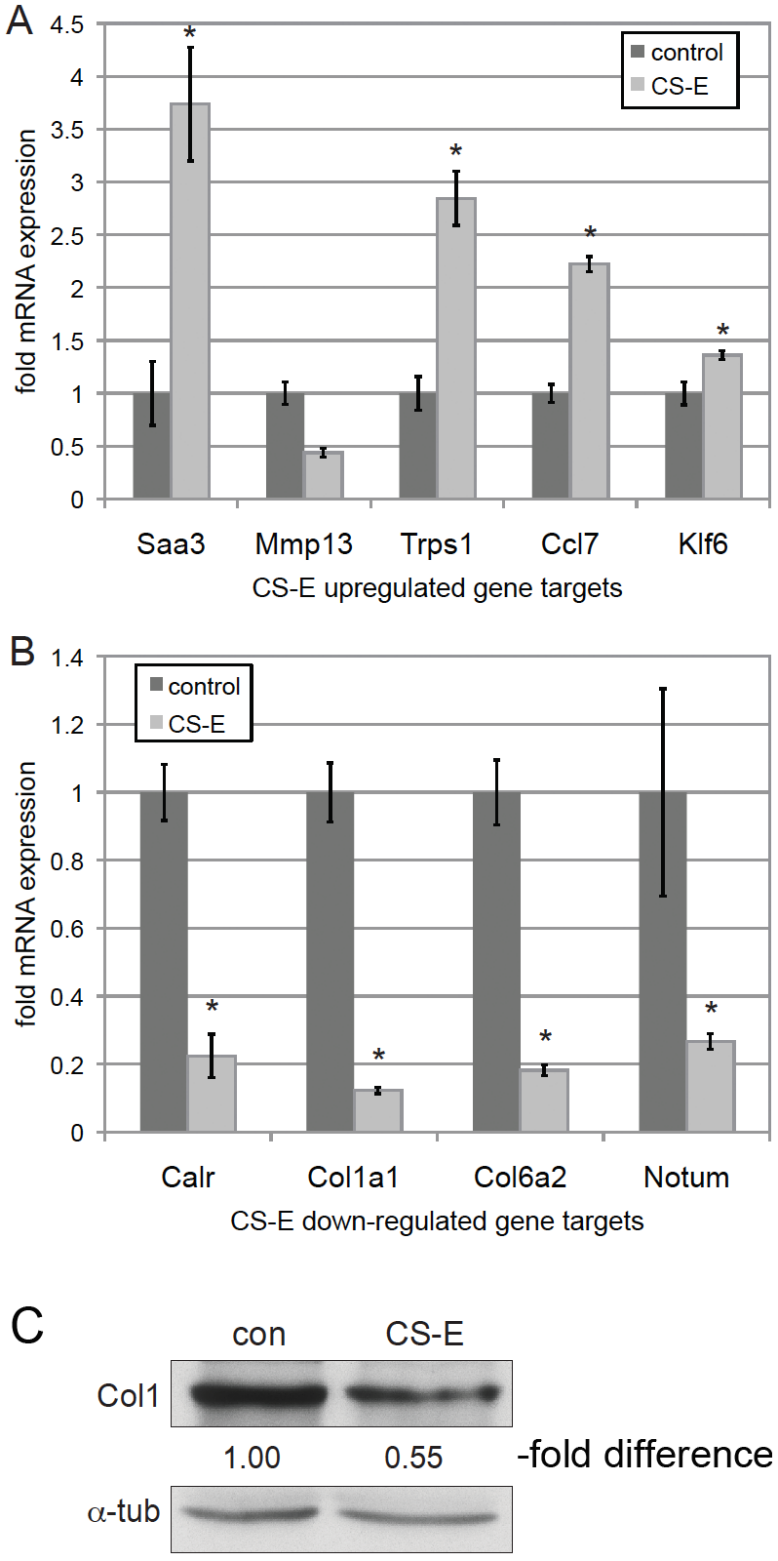
Confirmation of micoarray results: CS-E negatively regulates Col1a1 mRNA and protein levels. (**A, B**) EMT6 cells were treated with or without 100 µg/ml CS-E for 6 days in 3D on-top Matrigel culture assays, followed by RNA preparation and qRT-PCR. (**A**) Evaluation of effects of CS-E treatment on expression of positively-regulated target genes Saa3, Mmp13, Trps1, Ccl7, and Klf6. (**B**) Evaluation of effects of CS-E treatment on expression of negatively-regulated target genes Calr, Col1a1, Col6a2, and Notum. (*p<0.05). (n = 3). (**C**) Immunoblot of type 1 collagen protein levels (Col1) of EMT6 3D structures treated with or without CS-E. Treatment with CS-E reduced Col1 protein levels to 55% when corrected for alpha-tubulin loading controls and compared to untreated control cultures (con).

Next, we set out to characterize the mechanism by which CS-E elicits the inhibitory effect observed on the invasive protrusion formation of EMT6 3D structures. First, we investigated whether CS-E treatment changed the percentage of proliferating cells, or the percentage of cells undergoing apoptosis in EMT6 3D structures. For proliferation studies, EMT6 3D cultures were treated with or without CS-E, and EdU was incorporated at Day 6 of culture. The percentage of EdU-positive cells was quantified by co-staining with DAPI. There was no significant difference in the percentage of proliferating cells in EMT6 3D structures when treated with CS-E compared to control conditions (Figure 1C). To evaluate apoptosis in EMT6 3D cultures, we utilized TUNEL staining protocols at day 6 of culture. The percentage of TUNEL-positive cells was quantified by co-staining with DAPI. We detected no significant levels of cellular apoptosis in either the presence or absence of CS-E (data not shown). We then asked whether CS-E had any effect on the migratory or invasive potential of EMT6 cells (Figure 1D). For this, EMT6 cells were plated on either migration or invasion trans-well inserts in the presence or absence of CS-E for 24 hours. CS-E treatment was able to reduce cell migration rates by about 2.3-fold when compared to untreated controls (Figure 1D). In contrast, CS-E had no effect on percent cell invasion in these assays (Figure 2E). These results demonstrate that CS-E treatment was able to significantly inhibit the migratory abilities of EMT6 cells.

### Effects of CS-E treatment on EMT6 gene expression profiles

Given the intriguing function of CS-E in EMT6 cell behavior, we next wanted to determine CS-E-mediated gene expression profiles by microarray analysis. For this, EMT6 cells were grown in 3D on-top Matrigel assays and treated in the presence or absence of CS-E at 100 microgram/ml. Cells were harvested for RNA preparation on day 6 of culture. Gene expression profiles were analyzed on IlluminaMouseBead chips. This analysis identified 18 genes with an increase in expression of 1.8-fold or more with CS-E treatment, and 12 genes with a decrease in expression of 1.8-fold or more (Table 1). To confirm these results, we quantified the effects of CS-E treatment on expression of five upregulated genes (Serine amyloid A 3 [Saa3], Trichorhinophalangeal syndrome I gene [Trps1], Chemokine (C-C motif) ligand 7 [Ccl7], Krueppel-like factor 6 [Klf6] and Matrix-metaloproteinase 13 [Mmp13]), and four downregulated genes (Calreticulin [Calr], Collagen, type 1, alpha 1 [Col1a1], Collagen, type 6, alpha 2 [Col6a2], and Notum [Notum]), by quantitative Reverse Transcription-PCR (qRT-PCR) (Figure 2A and 2B). These results confirmed the effects of CS-E on the expression of the identified target genes, with the exception of Mmp13, which appeared not to be upregulated by CS-E (Figure 2A). Taken together, our results identify a novel CS-E-mediated gene expression signature in EMT6 breast cancer cells.

**Table 1:** Microarray results of the effects of CS-E on gene expression profiles of EMT6 3D structures.

		(fold-changes in mRNA expression)
Gene Symbol	Gene Title Annotation	Induction by CS-E (CS-E/control)
Saa3	serine amyloid A 3	4.22
Mmp13	matrix-metaloproteinase 13	2.82
Trps1	trichorhinophalangeal syndrome I gene	2.18
Ccl7	chemokine (C-C motif) ligand 7	2.16
Fam134b	family with sequence similarity 134, member B	2.11
Klf6	krueppel-life factor 6	2.07
Tlr2	toll-like receptor 2	2.01
Rhbdl2	rhomboid-like protein 2	1.95
Styx	serine/threonine/tyrosine interacting protein	1.95
Mgp	matrix Gla protein	1.92
Chka	choline kinase alpha	1.92
Gdpd1	glycerophosphodiester phosphodiesterase	1.92
	domain containing 1	
Mrps15	mitochondrial ribosomal protein S15	1.89
Atp6v1a	ATPase, H+ transporting, lysosomal	1.89
	70kDa, V1 subunit A	
Zxda	zinc finger, X-linked, duplicated A	1.88
BC030476	cDNA sequence BC030476	1.83
Cd68	CD68 molecule, macrosialin	1.82
Socs2	suppressor of cytokine signaling 2	1.81
**Gene Symbol**	**Gene Title Annotation**	**Repression by CS-E (CS-E/control)**
Calr	calreticulin	–2.36
Col1a1	collagen, type 1, alpha 1	–2.18
Gnai2	guanine nucleotide binding protein (G protein),	–2.01
	alpha inhibiting activity polypeptide 2	
Sept5	septin 5	–1.98
Col6a2	collagen, type 6, alpha 2	–1.97
Ankrd37	ankyrin repeat domain 37	–1.93
Notum	notum pectinacetylesterase homolog	–1.9
Pfn1	profilin 1	–1.9
Csrp2	cysteine and glycine-rich protein 2	–1.82
LOC100047583	apolipoprotein D-like	–1.81
Tmsb10	thymosin beta 10	–1.8
Asna1	arsA arsenite transporter, ATP-binding, hom.1	–1.8

Shown are fold-changes in mRNA expression (≥1.8-fold induction/repression). Two pro-tumorigenic Collagen genes (*Col1a1*, *Col6a2*) were repressed by CS-E treatment.

### Downregulation of Col1a1 expression is required for the anti-migratory effects of CS-E in two different breast cancer cell lines

Our microarray results revealed two collagen target genes that were repressed by CS-E treatment. Collagens are major components of the tumor-stromal environment, and play important roles in cancer cell behavior. Increased extracellular levels of Col1a1 have been shown to promote tumor cell invasiveness in culture and metastasis in animal models. Similarly, a high level of Col1a1 has been associated with an increased likelihood of clinical metastasis of multiple human solid tumors [33]–[35]. We therefore hypothesized that CS-E treatment interfered with invasive protrusion formation and cell motility by negative regulation of Col1a1 expression. We first wanted to determine whether the repression of *Col1a1* gene expression by CS-E translates into reduced protein levels. For this, Col1a1 protein expression was quantified from whole cell protein lysates from EMT6 and 3D on-top Matrigel cultures (Figure 2C). Immunoblotting with an anti-type 1 collagen antibody (Col1) showed that CS-E treatment reduces the level of type 1 collagen protein by about 50% compared to controls.

**Figure 3:**
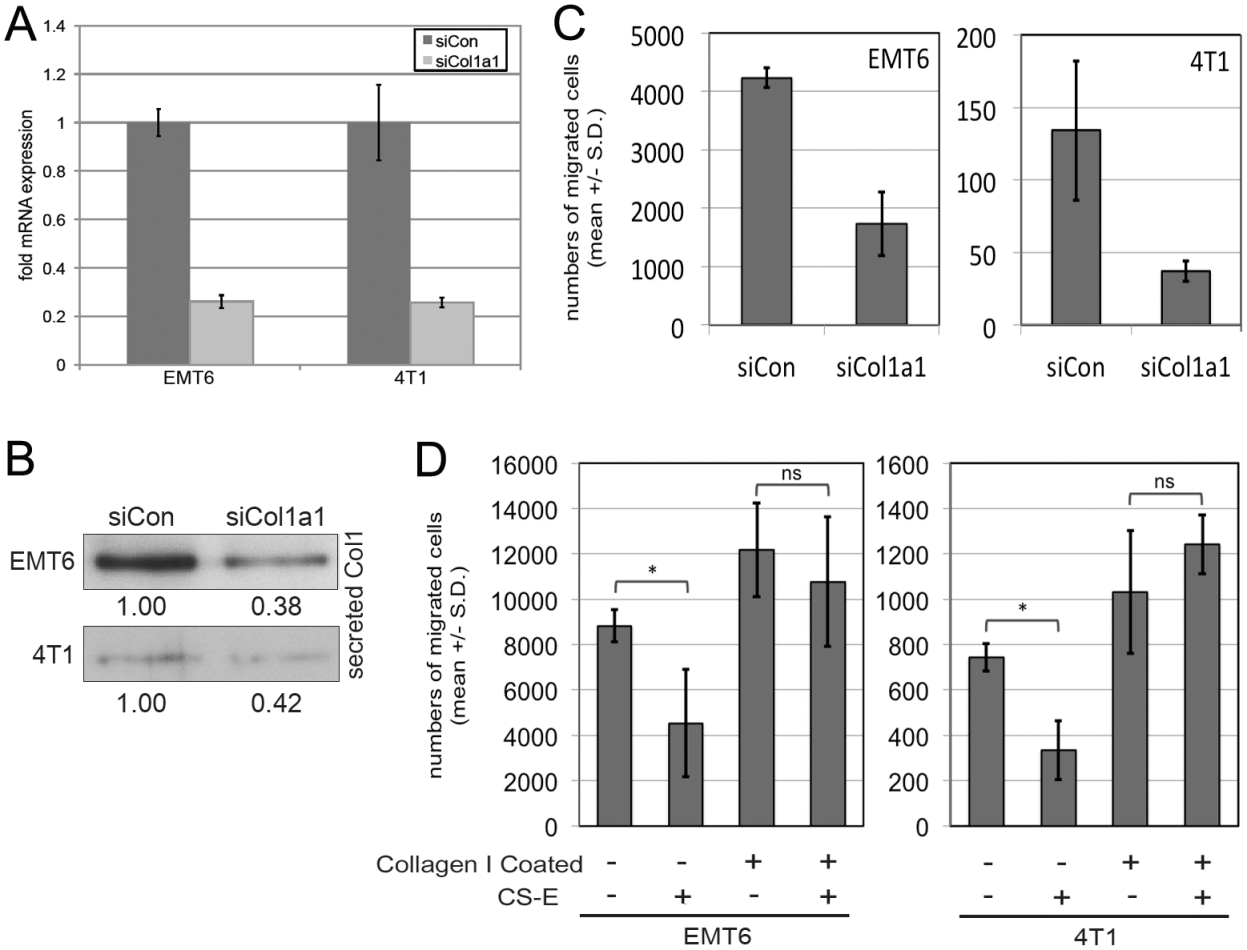
Negative regulation of Col1a1 expression by CS-E is required for inhibition of cell migration in EMT6 and 4T1 breast cancer cells. (**A**) Knockdown of Col1a1: *Col1a1* mRNA levels were compared by qRT-PCR after 24 hrs of either Col1a1 siRNA (siCol1a1) or non-targeting siRNA control (siCon) knockdown. Col1a1 siRNA knockdown reduced *Col1a1* expression to approximately 25% in both EMT6 and 4T1 cells. (**B**) Western blot analysis of the effect of siCol1a1 or siCon on secreted Col1 protein levels. Transfection of siCol1a1 reduces levels of secreted type 1 collagen in both EMT6 and 4T1 cells. (**C**) siRNA knockdown of Col1a1 significantly reduces breast cancer cell motility in 24 hour transwell experiments. (**D**) Establishing an exogenous collagen I matrix interfered with the inhibitory effects of CS-E on cell migration. EMT6 or 4T1 cells were plated onto migration inserts that were uncoated or coated with purified bovine type 1 collagen and were treated with or without 100 microgram/ml CS-E for 8 hours in a transwell migration assay. CS-E could interfere with cell migration in the absence, but not the presence of an exogenously supplied collagen I matrix. Graph shows average number of migrating cells per experiment (n = 3). (*p<0.05; ns = not significant).

The following studies were performed on EMT6 as well as 4T1 mouse breast cancer cell lines, in order to ensure that the effects observed are not cell line-specific. We asked whether the CS-E-mediated reduction in Col1a1 gene and protein expression is functionally important in the phenotypes we see with exogenous CS-E treatment. If this were to be the case, we would expect that a loss of Col1a1 expression would mimic the effects observed with CS-E. To address this question, we tested whether siRNA knockdown of *Col1a1* could efficiently reduce Col1a1 expression, and whether this treatment could affect cell migration (Figure 3A–C). First, we determined that a *Col1a1* siRNA (siCol1a1) efficiently knocked-down transcript (Figure 3A) and secreted protein levels (Figure 3B) compared to the non-targeting control siRNA (siCon). Having confirmed the functionality of our *Col1a1* siRNA, we then analyzed its effects on EMT6 and 4T1 cell migration. Cells were transfected with siCon or siCol1a1, and cell migration was analyzed in transwell assays. Knock-down with siCol1a1 reduced the number of migrating cells 2.5 fold (EMT6) and 3-fold (4T1) (Figure 3C). Thus, we show that Col1a1 is a positive regulator of breast cancer cell migration, and that inhibition of Col1a1 expression could mimic the effects of CS-E treatment.

Next, we wanted to determine whether exogenously supplying a type 1 collagen matrix could overcome the negative effects of CS-E on Cola1a expression and rescue the migration defects seen with CS-E treatment. We performed transwell migration assays on uncoated 8 µm pore membranes, or membranes coated with 500 microgram/ml bovine type 1 collagen on the outer-side of membranes. Cells were treated with or without 100 microgram/ml CS-E on both uncoated and coated migration inserts for 8 hours. Treatment with CS-E was able to significantly interfere with the migration of EMT6 and 4T1 cells on uncoated membranes (Figure 3D). Supplying an extracellular collagen matrix by coating membranes with type 1 collagen led to an increase in cell migration (Figure 3D). However, CS-E treatment of breast cancer cells plated on type 1 collagen-coated membranes was now unable to interfere with cell migration (Figure 3D), demonstrating that an exogenous Collagen matrix could overcome the CS-E-mediated negative regulation of Col1a1 expression and cell migration. Together, these data show that CS-E could interfere with expression of type 1 collagen, a positive regulator of breast cancer cell migration. Moreover, the negative regulation of *Col1a1* gene expression by CS-E is necessary for its inhibitory effect on breast cancer cell motility.

### CS-E interferes with Wnt/beta-catenin signaling in breast cancer cells

**Figure 4:**
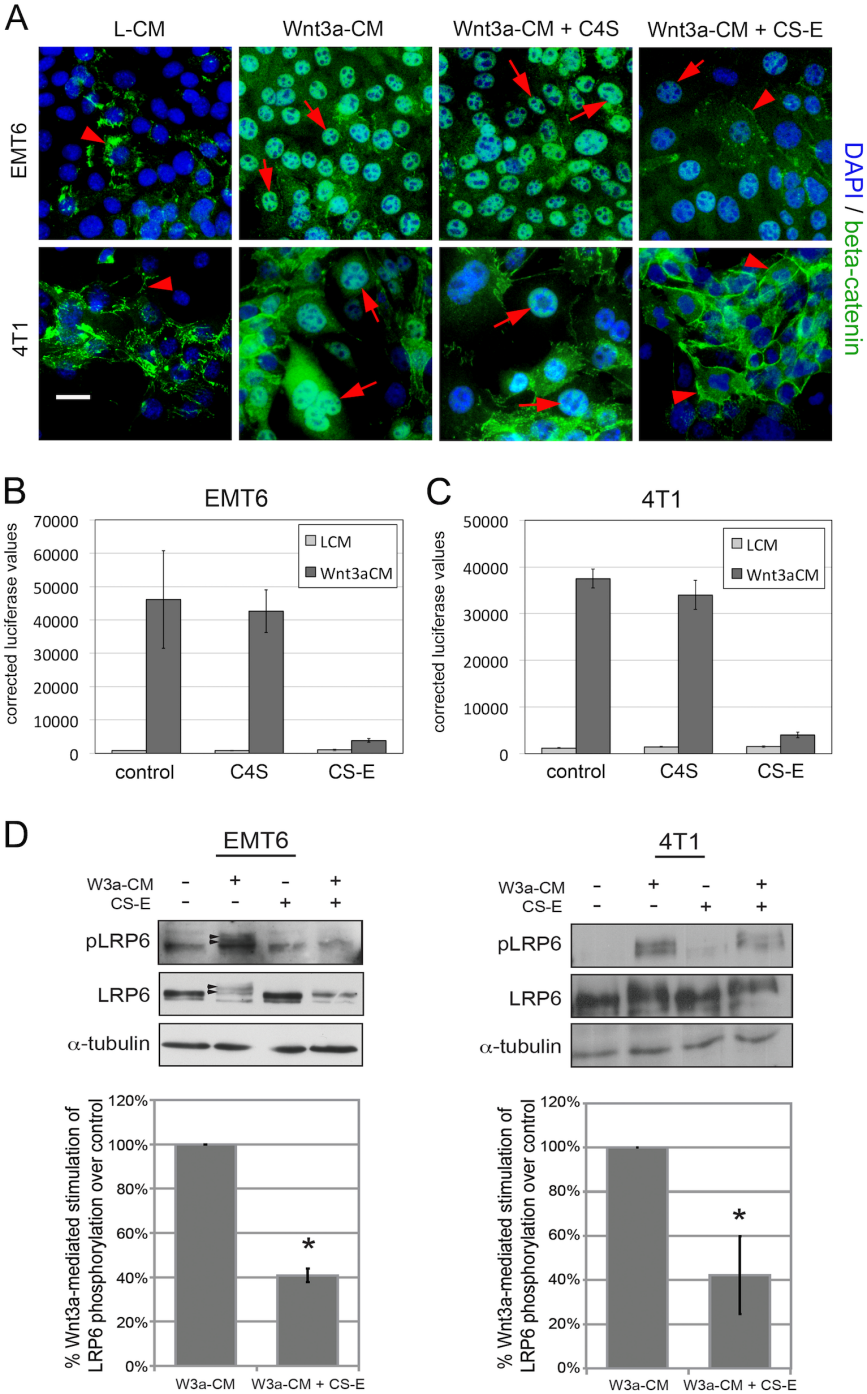
CS-E inhibits Wnt/beta-catenin signaling in EMT6 and 4T1 breast cancer cells at the cell surface receptor level. (**A**) Immuno-fluorescence detection of beta-catenin in 2D monolayers of EMT6 or 4T1 cells treated with L-CM or Wnt3a-CM in the presence or absence of C4S or CS-E treatment. Wnt3a-CM led to a drastic increase in nuclear beta-catenin levels; concomitant treatment with CS-E, but not C4S, severely reduced nuclear beta-catenin expression levels (red arrowheads: cell membrane staining; red arrows: nuclear staining) (beta-catenin: green; DAPI: blue; scale bar = 50 micrometer). (**B, C**) Effect of CS-E on TOPFLASH reporter assays. Treatment with Wnt3a-CM for 24 hours caused a significant increase in TOPFLASH luciferase activity when compared to treatment with L-CM in both EMT6 (**B**) and 4T1 cells (**C**). Concomitant treatment with CS-E, but not C4S, significantly reduced Wnt3a-stimulated TOPFLASH reporter activity. (**D**) Western blot analysis of Wnt/beta-catenin signaling receptor activation. Treatment of EMT6 cells with Wnt3a-CM, but not L-CM for 1 hour led to phosphorylation of LRP6 (pLRP6). Concomitant treatment with CS-E (100 microgram/ml) interfered with phosphorylation of the LRP6 protein. Note that only the upper bands of the LRP6 are phosphorylated in response to Wnt3a (black arrows). Quantitation of these Western blots demonstrated that CS-E treatment significantly reduced LRP6 receptor phosphorylation by 60% in both EMT6 and 4T1 cells (*p<0.01; n≥3).

We have shown that treatment with exogenous CS-E interfered with breast cancer cell motility through negative regulation of the expression of the pro-tumorigenic ECM molecule Col1a1. We and others have previously identified an important role of CS-E in the control of the pro-tumorigenic canonical Wnt/beta-catenin pathway [7], [11], [13], [36], [37]. Thus, we set out to determine whether the effects of CS-E on cell migration and collagen1a1 expression are mediated by Wnt/beta-catenin. First, we analyzed the effects of CS-E on expression and subcellular localization of beta-catenin in the presence of control conditioned media (L-CM) or Wnt3a conditioned media (Wnt3a-CM) by immunofluorescence (Figure 4A). As a negative control, we treated cells with C4S, which has previously been shown not to modulate Wnt/beta-catenin signaling [8], [32], [34]. In the absence of Wnt3a stimulation (L-CM), beta-catenin levels were low and observed mostly in cell membranes in both EMT6 and 4T1 cells (Figure 4A). Treatment with Wnt3a-CM led to a drastic increase in beta-catenin levels and nuclear translocation in both cell types (Figure 4A). Addition of C4S did not alter the Wnt3a-stimulated nuclear accumulation of beta-catenin, consistent with the inability of C4S to inhibit Wnt/beta-catenin signaling (Figure 4A). However, addition of CS-E treatment to Wnt3a stimulation severely reduced nuclear beta-catenin expression levels (Figure 4A), suggesting that CS-E, but not C4S, is a potent inhibitor of Wnt/beta-catenin signaling in breast cancer cells.

Next, we performed TOPFLASH luciferase reporter assays to evaluate the effect of CS-E, or C4S as a negative control, on beta-catenin transcriptional readout. Treatment of EMT6 or 4T1 cells with CS-E severely inhibited Wnt3a-stimulated TOPFLASH activity, while C4S treatment had no effect on TOPFLASH activity (Figure 4B, C). Overall, these data show that CS-E, but not C4S, can negatively regulate Wnt/beta-catenin signaling in our breast cancer cells. CS-E has previously been show to bind Wnt3a ligand with high affinity [34], and we have shown that CS-E treatment leads to reduced Wnt3a receptor complex activation in fibroblasts [8]. Thus, we wanted to determine whether CS-E could interfere with Wnt3a-mediated receptor activation in breast cancer cells. For this, we analyzed the phosphorylation of the Wnt/β-catenin co-receptor LRP6 (Figure 4D). Western blot analysis of EMT6 or 4T1 whole cell lysates showed that non-stimulated cells presented one band for LRP6, while no bands were detected with the phospho-LRP6 antibody (Figure 4D). Upon Wnt3a-stimulation for 1 hour, the majority of LRP6 became phosphorylated and shifted to two phosphorylated pLRP6 products, with an according reduction in the levels of unphosphorylated LRP6 (Figure 4D). Of note, a negative feedback loop, in which Wnt signaling negatively regulates arrow/LRP expression, has been described in Drosophila [38]. A potential contribution of this potential feedback loop to regulation of LRP6 expression in breast cancer cells is currently unknown. Concomitant treatment with CS-E interfered with Wnt3a-stimulated phosphorylation of LRP6, as illustrated by reduced pLRP6 levels in both EMT6 and 4T1 cells (Figure 4D). Quantification of the effects of CS-E on Wnt3a-stimulated receptor activation demonstrated a 60% reduction in pLRP6 levels in the presence of CS-E (Figure 4D). Together, these data show that CS-E is a *bona fide* inhibitor of the Wnt/beta-catenin signaling pathway in EMT6 and 4T1 breast cancer cells, and that CS-E treatment interferes with Wnt3a-stimulated receptor activation at the cell surface.

### *Col1a1* is a Wnt/beta-catenin target gene in breast cancer cells

**Figure 5:**
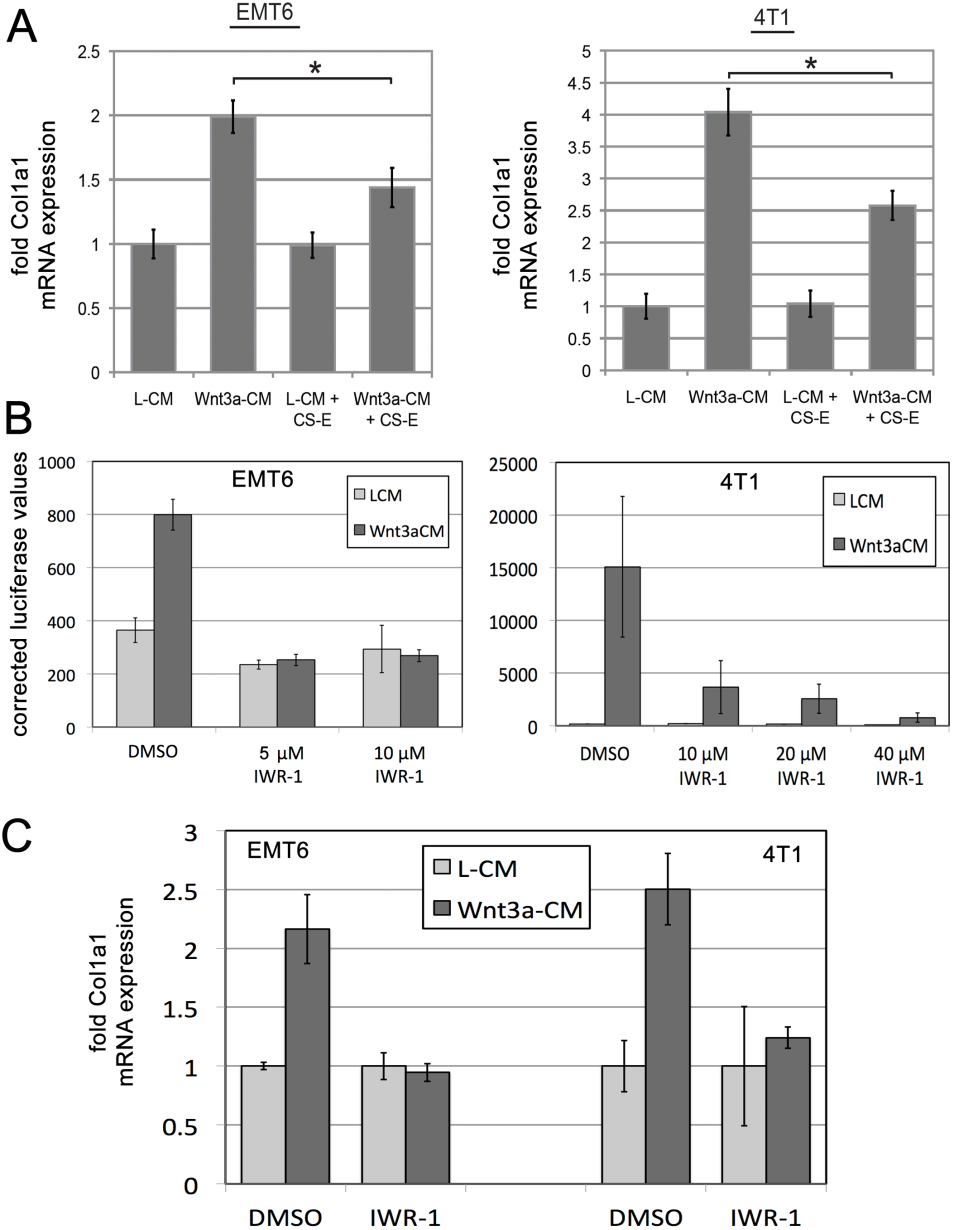
*Col1a1* is a Wnt/beta-catenin target gene in breast cancer cells. (**A**) RTqPCR to quantify *Col1a1* mRNA expression in response to Wnt3a stimulation, in the presence or absence of CS-E. Treatment with Wnt3a-CM led to a 2-fold (EMT6) and 4-fold (4T1) increase in *Col1a1* mRNA expression. Concomitant treatment with CS-E significantly interfered with this stimulatory effect of Wnt3a and reduced *Col1a1* expression levels (*p<0.05). (**B**) Wnt/beta-catenin pathway inhibition by IWR-1. IWR-1 at both 5 microMolar and 15 microMolar completely inhibited Wnt3a-stimulated TOPFLASH activity to the level of control treated cells in EMT6 cells. In 4T1 cells, an IWR-1 concentration of 40 microM led to an almost complete loss of TOPFLASH activity. (**C**) RTqPCR analysis: inhibition of Wnt/beta-catenin signaling by IWR-1 (EMT6 cells: 5 microMolar; 4T1: 40 microMolar) led to a loss of Wnt3a-mediated induction of *Col1a1* mRNA expression in EMT6 and 4T1 cells.

Our data so far led us to hypothesize that interference with Wnt/beta-catenin signaling by CS-E treatment leads to a loss of Col1a1 expression. Next, we wanted to identify *Col1a1* as a target gene of Wnt3a signaling. For this, we utilized RTqPCR to quantify *Col1a1* mRNA expression in response to Wnt3a stimulation, in the presence or absence of CS-E (Figure 5A). Treatment with Wnt3a-CM led to a 2-fold (EMT6) and 4-fold (4T1) increase in *Col1a1* mRNA expression (Figure 5A), showing that *Col1a1* expression is positively regulated by Wnt/beta-catenin signaling. Concomitant treatment with CS-E significantly interfered with this stimulatory effect of Wnt3a and reduced *Col1a1* expression levels (Figure 5A).

Next, we wanted to determine whether inhibition of Wnt/beta-catenin signaling by other means has a similar effect on *Col1a1* expression. For this, we utilized Inhibitor-of-Wnt-Response-1 (IWR-1), an established small molecule inhibitor of Wnt/beta-catenin signaling [39]. First, we established the correct concentrations of IWR-1 to inhibit the Wnt/beta-catenin pathway by utilizing the TOPFLASH luciferase reporter assay. EMT6 or 4T1 cells were treated with DMSO as a vehicle control, and various concentrations of IWR-1 in the presence of L-CM or Wnt3a-CM for 24 hours and assayed for TOPFLASH activity (Figure 5B). IWR-1 at both 5 and 15 microMolar completely inhibited Wnt3a-stimulated TOPFLASH activity to the level of control treated cells in EMT6 cells. In 4T1 cells, we observed a dose-response curve with almost complete loss of TOPFLASH activity at 40 microMolar (Figure 5B). We then wanted to determine whether interference with Wnt3a signaling by treatment with IWR-1 at 5 microMolar (EMT6) and 40 microMolar (4T1) would affect *Col1a1* expression. Indeed, RT-qPCR experiments demonstrated that inhibition of Wnt/beta-catenin signaling by IWR-1 led to a loss of Wnt3a-mediated induction of *Col1a1* mRNA expression (Figure 5C). Together, these data established a Wnt/beta-catenin - collagen axis in breast cancer cells, and demonstrated that Wnt/beta-catenin pathway inhibition by either IWR-1 or CS-E interferes with Wnt3a-mediated induction of *Col1a1* expression.

## Discussion

Here, we describe the functional and mechanistic role of CS-E in metastatic breast cancer cell behavior. We show that CS-E, but not other chondroitin forms, could interfere with the invasive protrusion formation of highly metastatic murine breast cancer cells in 3D organotypic cultures. We further demonstrate that negative regulation of *Cola1a* gene expression by CS-E treatment was required for its anti-migratory effects. CS-E interfered with Wnt/beta-catenin signaling, a known pro-tumorigenic pathway. We further established that *Col1a1* is a positively regulated target gene of the Wnt/beta-catenin pathway in breast cancer cells. Together, our data demonstrate that CS-E could negatively regulate *Col1a1* gene expression through inhibition of Wnt/beta-catenin signaling, which in turn led to decreased breast cancer cell motility. These data identify a novel chondroitin sulfate-based control mechanism for a Wnt/beta-catenin-collagen pro-tumorigenic axis in organotypic cell cultures. Based on our results, it will be interesting to investigate whether similar roles exist for endogenous tumor-associated CS-E molecules in Wnt/beta-catenin signaling, Collagen I expression, and breast cancer progression *in vivo*, and to investigate a potential therapeutic use of CS-E as an inhibitor of the pro-tumorigenic Wnt/beta-catenin/Collagen I pathway in breast cancer.

A significant increase in the deposition of chondroitin sulfate proteoglycans has been observed in the microenvironment of several human solid tumors, including breast cancer [16]–[20]. We have recently shown that enzymatic elimination of chondroitin sulfate molecules in primary tumors in mice carrying orthotopic breast tumors lead to an increase in lung metastases [11]. Conversely, digestion of cell surface chondroitin sulfate molecules on cancer cells injected into tail veins lead to reduced numbers of tumor cells able to populate and form metastases in target organs [24]. Furthermore, Monzavi-Karbassi et al. [20] have shown that chondroitin sulfate side chains on the cell surface of breast cancer cells facilitate the interaction with Selectin proteins on endothelial cells *in vitro*. Together, these data suggest that chondroitin sulfate molecules can have temporally op­posing functions during cancer progression: an anti-metastatic function in primary tu­mor tissue, but a pro-metastatic role during the interaction of circulating cancer cells with endothelial cells (extravasation). However, a molecular mechanism by which CS could influence breast cancer cell behavior in primary tumors had not been elucidated. Our work here indicates that CS-E-mediated inhibition of the pro-tumorigenic Wnt/beta-catenin pathway in breast cancer cells provides one molecular mechanism by which chondroitin sulfates control Cola1a1 expression and alter a pro-tumorigenic breast cancer cell microenvironment. In future studies, it will be interesting to investigate the status of endogenous Wnt/beta-catenin signaling and Col1a1 levels in tumors in which chondroitin sulfate molecules have been eliminated.

While our results describe a novel CS-E-mediated upstream regulatory mechanism for Wnt/beta-catenin signaling and expression of pro-tumorigenic collagen proteins, the analysis of signaling events downstream of this cascade is also of great interest. Cell movements *in vivo* usually require integrin function to facilitate cell-ECM interactions. Integrins can interact with type I collagens in the ECM and initiate critical signaling cascades [40]; therefore, it will be interesting to investigate how the repression of pro-tumorigenic collagen genes by CS-E influences integrin-collagen interactions and signaling events downstream of integrin cell surface receptors.

Given the drastic effects of CS-E on breast cancer cell behavior and Wnt/beta-catenin signaling, we were surprised by the relatively small number of genes affected by CS-E treatment. Genome-wide microarray studies have established much larger Wnt gene signatures in many cancers, including breast cancer, and other cell types [13], [41], [42]. One possible explanation for these observations could be that CS-E might not affect all Wnt/beta-catenin target genes. Indeed, in a recent study we have shown that CS-E treatment of NIH3T3 fibroblasts could inhibit positively regulated, but not negatively-regulated Wnt3a target genes [13]. Moreover, in the same study we demonstrated that CS-E treatment reduced Wnt3a signaling to a critical threshold that dissociated molecular and biological readouts of Wnt/beta-catenin pathway activation. It is tempting to speculate that CS-E treatment of breast cancer cells might identifiy similar Wnt/beta-catenin signaling thresholds, and therefore only affects a subset of Wnt/beta-catenin target genes and/or biological readouts.

Overall, our data in this and previous studies [17], [32] suggested that CS-E can play a role in the fine-tuning of Wnt/beta-catenin signaling. In this context, it is of interest to note that specific, but not necessarily extreme, levels of Wnt/beta-catenin signaling have been suggested to be advantageous in tumor progression, development, stem cell renewal, and tissue maintenance [43], [44]. For example, the “just right” signaling model of Wnt/beta-catenin signaling in familial adenomatous polyposis (FAP) demonstrated a selection for genetic mutations in the *APC* gene that retain some of its ability to decrease β-catenin signaling levels, rather than mutations that result in complete loss of function of APC and subsequent constitutive Wnt/beta-catenin signaling [45]. Since activating mutations in core components of the Wnt/beta-catenin pathway are rarely found in breast cancer, we hypothesize the existence of a complex network of positive and negative regulatory mechanisms that establish precise Wnt/beta-catenin signaling levels most beneficial for a preferred balance of tumor growth, survival, and overall progression. While several activating mechanism have been identified (i.e. loss of expression of extracellular inhibitors), we propose that chondroitin sulfate in the tumor microenvironment, including CS-E, participates in this complex regulatory network of the Wnt/beta-catenin pathway.

This work has important pharmacological aspects. Aberrantly active Wnt/beta-catenin signaling has been implicated in many tumors, including human breast cancers, and this has been correlated with poor prognosis for patients [24], [25]. The identification and characterization of novel mechanisms to target the Wnt/beta-catenin signaling pathway has become an area of intense study in the breast cancer research field. Our data identify a novel chondroitin sulfate-based control mechanism for a Wnt/beta-catenin-Collagen pro-metastatic axis, and provide evidence for a potential therapeutic use of CS-E treatment as an inhibitor of Wnt/beta-catenin signaling in breast and other cancers.

## Supporting Information

Figure S1 Specificity of CS-E in the inhibition of invasive protrusion formation. **(A)** EMT6 Matrigel 3D cultures. **(B)** Quantitation of invasive protrusions. EMT6 cells were grown in Matrigel 3D cultures. Treatment with CS-E at 100 microgram/ml for 6 days lead to an almost complete inhibition of protrusion formation. Digestion of CS-E with Chondroitinase ABC for 2 hours prior to addition to EMT6 cultures eliminated this effect. Treatment of 3D cultures for 6 days with C4S, C6S, or CS-D at 500 microgram/ml lead to a small decrease in the percent of 3D structures with 5 or more invasive protrusions (*p<0.05; ns = not significant).
(TIFF)
