# Supplementary figures and images for: Chondroitin Sulfate-E Is a Negative Regulator of a Pro-Tumorigenic Wnt/Beta-Catenin-Collagen 1 Axis in Breast Cancer Cells
Source: PLoS One. 2014 Aug 4;9(8):e103966. doi: 10.1371/journal.pone.0103966 (PMC4121171; doi:10.1371/journal.pone.0103966)

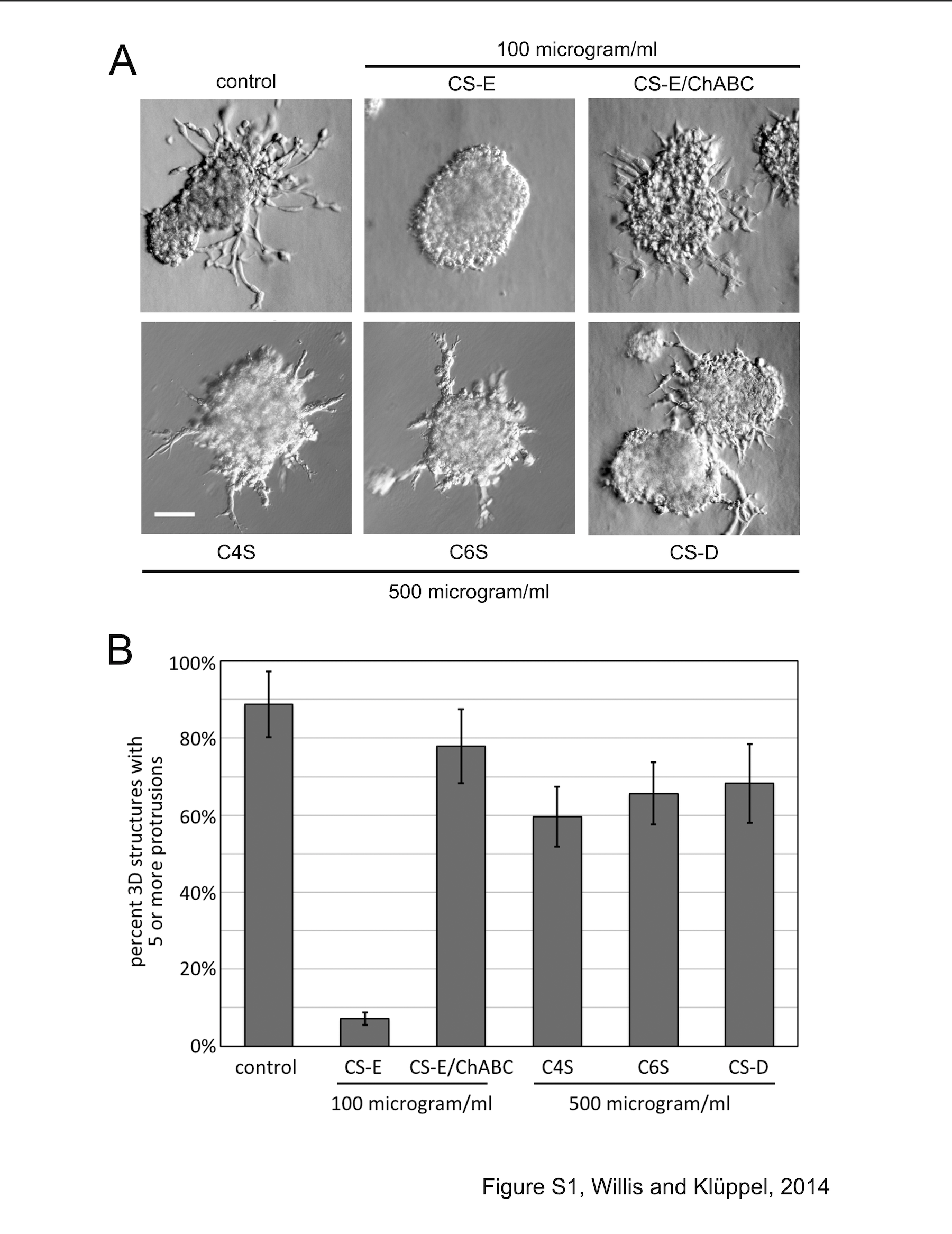

Supplement: Figure S1 — Specificity of CS-E in the inhibition of invasive protrusion formation. (A) EMT6 Matrigel 3D cultures. (B) Quantitation of invasive protrusions. EMT6 cells were grown in Matrigel 3D cultures. Treatment with CS-E at 100 microgram/ml for 6 days lead to an almost complete inhibition of protrusion formation. Digestion of CS-E with Chondroitinase ABC for 2 hours prior to addition to EMT6 cultures eliminated this effect. Treatment of 3D cultures for 6 days with C4S, C6S, or CS-D at 500 microgram/ml lead to a small decrease in the percent of 3D structures with 5 or more invasive protrusions (*p<0.05; ns = not significant). (TIFF) [file pone.0103966.s001.tif]
